# Impact of the COVID-19 pandemic on the quality of life of early breast cancer patients undergoing adjuvant chemotherapy – an observational, multicenter study

**DOI:** 10.2340/1651-226X.2025.43691

**Published:** 2025-10-08

**Authors:** Marie Tuomarila, Paula Poikonen-Saksela, Haridimos Kondylakis, Johanna Mattson, Päivi Auvinen, Arja Jukkola, Ilja Kalashnikov, Jussi Koivunen, Okko-Sakari Kääriäinen, Kaisa Sunela, Meri Utriainen, Pia Vihinen, Sirpa Leppä, Peeter Karihtala

**Affiliations:** a Department of Oncology, Helsinki University Hospital Comprehensive Cancer Center and University of Helsinki, Helsinki, Finland; b Institute of Computer Science, Foundation for Research and Technology-Hellas, Heraklion, Greece; c Cancer Center, Department of Oncology, Kuopio University Hospital, Kuopio, Finland; d Department of Oncology, Tampere University Hospital Cancer Center, Tampere, Finland; e Research Program Unit, Applied Tumor Genomics, Faculty of Medicine, University of Helsinki, Helsinki, Finland; f Department of Oncology and Radiotherapy, Oulu University Hospital and MRC Oulu, Oulu, Finland; g FICAN West Cancer Centre, Turku University Central Hospital, Turku, Finland

**Keywords:** Breast cancer, COVID-19 pandemic, health-related quality of life, HRQoL, EORTC QLQ-C30, adjuvant chemotherapy

## Abstract

**Background and purpose:**

We evaluated the impact of the coronavirus disease 2019 (COVID-19) pandemic on health-related quality of life (HRQoL) in early-stage breast cancer patients receiving adjuvant chemotherapy.

**Patients and methods:**

The study involved 180 patients with stage I–III breast cancer who initiated adjuvant chemotherapy between June 2020 and May 2021. The pre-pandemic comparison data included 113 early breast cancer patients who began adjuvant chemotherapy between November 2018 and August 2019. HRQoL was assessed using the EORTC QLQ-C30 at baseline and again after 3 and 6 months. The subscales were compared between the COVID-19 pandemic and the pre-pandemic eras.

**Results:**

We observed deterioration on almost all HRQoL subscales of the patients treated during the pandemic from baseline to 3 months. After the chemotherapy at 6 months, the scales remained deteriorated, whereas only appetite loss and emotional functioning improved. A comparison between the pandemic and the pre-pandemic eras revealed that several HRQoL subscales showed better results during chemotherapy in the pandemic era. Global health and role functioning at 6 months presented declined levels during the pandemic.

**Interpretation:**

The well-being of breast cancer patients during the chemotherapy treatment in the pandemic era was moderately better than in the pre-pandemic era. Patients in the pandemic era might have reported fewer symptoms during the treatment, as the focus was on the COVID-19 pandemic and its restrictions.

## Introduction

The coronavirus disease 2019 (COVID-19) pandemic caused an extreme burden on cancer care worldwide. As cancer patients are more vulnerable to COVID-19 infections than healthy persons, a higher number of adverse effects and death rates were observed [1]. Cancer care was compromised during the pandemic, and the cancer screening programs, including mammographic screening, were reduced [2], and delays and cancellations in planned cancer surgery and changes to medical therapy were observed [3–5].

Breast cancer is the most common malignancy in women [6]. Adjuvant chemotherapy, usually administered for 18–24 weeks, significantly improves disease-free and overall survival in patients with early-stage breast cancer [6, 7]. However, the adjuvant chemotherapy for these patients negatively impacts their Health-related Quality of life (HRQoL) [8], as, for instance, the global health status and physical functioning tend to deteriorate during the chemotherapy [9–11]. Fatigue and insomnia are common during chemotherapy and can lower scores across all HRQoL functions [12]. The deterioration of the HRQoL may persist for up to 12–18 months, counting from the start of the treatment [13, 14]. Also, cognitive impairment [12, 15] and depression [16] affect many breast cancer patients during or after the chemotherapy. Various psychological symptoms, negative affect, coping reactions, a sense of control/ability to manage distressing conditions, social support and self-efficacy to cope with cancer are known predictors for HRQoL in early breast cancer patients [17]. These same factors are also important when coping with external stress factors such as a pandemic situation [18].

People living with and beyond cancer during the COVID-19 pandemic are vulnerable to adverse psychosocial reactions and outcomes [19]. Breast cancer patients had significantly worse scores in most HRQoL domains compared to controls during the COVID-19 pandemic, but the magnitude of the differences between the patients and controls remained similar [20]. Nevertheless, there is a lack of data on the HRQoL of breast cancer patients being treated with chemotherapy during the exceptional circumstances of the COVID-19 pandemic.

In this observational, multicenter study, we evaluated the impact of the COVID-19 pandemic on HRQoL in patients with stage I–III breast cancer receiving adjuvant chemotherapy. We measured HRQoL at three time points (baseline, 3 months and 6 months) and compared it with that of the pre-pandemic patients. To our knowledge, no such studies have been previously published.

## Materials and methods

### Study design and participants

This prospectively collected multicohort study included 180 patients diagnosed with early-stage breast cancer. Inclusion criteria for participating in the study were invasive, histologically confirmed breast cancer, intention to receive adjuvant chemotherapy, age >18 years, expected cooperation and signed informed consent.

Patients were recruited between the 1st of June 2020 and the 5th of May 2021 in the oncological departments of five Finnish Cancer Centers (Helsinki, Turku, Oulu, Kuopio and Tampere). Patients were offered participation during their first postoperative appointment by a treating oncologist.

The pre-pandemic reference data consisted of 113 Finnish breast cancer patients from Helsinki University Hospital Comprehensive Cancer Center and were part of a prospective, multicenter study, BOUNCE [21]. Inclusion criteria for the BOUNCE study were the presence of devoted informed consent, female sex, age 40–70 years at the time of the recruitment, histologically confirmed invasive early or locally advanced operable breast cancer, tumor stage I, II or III, patients receiving surgery as part of the local treatment and patients receiving any type of systemic treatment. Only patients who were diagnosed between the 23rd of November 2018 and the 1st of August 2019 and received adjuvant chemotherapy, were recruited, and all patients in this cohort had finished their 6-month follow-up period before the start of the COVID-19 pandemic, and were included as a comparison cohort.

All patients gave written consent before attempting the study. The regional ethics committee approved the study protocol (HUS/1377/2020).

### EORTC QLQ-C30

The European Organization for Research and Treatment of Cancer (EORTC) QLQ-C30 questionnaire [22], version 3.0, was used to assess the HRQoL. The self-reported paper questionnaire was completed three times, either at the hospital or at home: at the time of the diagnosis, within 14 days of recruitment and before the start of the chemotherapy (baseline), at three and at 6 months. The functional scales, the symptom scales and the global health status were scored from 0 to 100 according to the QLQ-C30 version 3.0 manual [23]. Higher scores for functional scales indicated better function, whereas higher scores in symptom scales implied worse symptoms.

The results from the COVID-19 era were compared with the pre-pandemic reference data from the BOUNCE study, where the EORTC-QLQ-C30 questionnaires were measured at identical time points. The missing answers within a questionnaire were excluded if less than half of the items from the scale were answered. Otherwise, the missing values were calculated according to the EORTC QLQ-C30 version 3.0 manual [23].

### Statistical analyses

To evaluate the associations of the clinical variables between the pandemic and the pre-pandemic patients, the two-sided chi-square test or Fisher’s exact test was used. Fisher’s exact test was used if the expected cell frequency was less than five. The Wilcoxon signed rank test was used to calculate the significant differences of HRQoL results at different time points for the patients treated during the pandemic. To calculate the statistically significant differences between the HRQoL results between the pandemic and the pre-pandemic groups, the Mann–Whitney *U* test was used. *P*-values less than 0.05 were considered statistically significant. All statistical analyses were performed using the IBM SPSS, version 28 (SPSS; IBM Corp., Armonk, NY).

## Results

### Patient demographics

Data from the pandemic era were collected from 174 (96.7% of all participating patients), 159 (88.3%) and 135 (75.0%) patients at the baseline, 3 and 6 months, respectively. Likewise, HRQoL data from the pre-pandemic era were available from 112 (99.1%), 106 (94.0%) and 104 (92.0%) patients at the baseline, 3 months and 6 months, respectively.

The baseline and treatment characteristics of the patients in the COVID-19 and pre-pandemic cohorts are shown in Table 1. The patients in the pandemic cohort had slightly higher BMI, were treated less frequently both with anthracyclines and taxanes and had more often HER2 -negative disease, reflecting the diverse distribution in the molecular subtype surrogates. Otherwise, baseline characteristics were equally distributed between the two cohorts.

**Table 1:** Clinical characteristics of the patients for the pandemic and pre-pandemic era.

Patient characteristics	Pandemic era	Pre-pandemic era	*p*
	Mean (SD), range	Mean (SD), range	
Age, *years*	57.8 (10.2), 36–82	55.5 (7.5), 41–68	0.072^1^
BMI *kg/m^2^*	28.5 (5.6), 19–46	26.5 (5.2), 18–43	**0.003** ^1^
	*n* (%)	*n* (%)	
Gender			1.00^3^
Female	179 (99.4)	113 (100)	
Male	1 (0.6)	0 (0)	
Treatment center			
Helsinki University Hospital	48 (26.7)	113 (100)	
Turku University Hospital	49 (26.7)		
Tampere University Hospital	23 (12.8)		
Kuopio University Hospital	32 (17.8)		
Oulu University Hospital	27 (15.0)		
Unknown	1 (0.6)		
Stage			0.461^2^
I	49 (27.2)	37 (32.7)	
II	91 (50.6)	63 (55.8)	
III	28 (15.6)	13 (11.5)	
Unknown	12 (6.7)	0 (0)	
Lymph node involvement			0.626^2^
N0	84 (46.7)	48 (42.5)	
N1	67 (37.2)	53 (46.9)	
N2	11 (6.1)	6 (5.3)	
N3	6 (3.3)	4 (3.5)	
Unknown	12 (6.7)	2 (1.8)	
Tumour size			0.079^2^
T1	85 (47.2)	66 (58.4)	
T2	67 (37.2)	41 (36.3)	
T3	14 (7.8)	3 (2.7)	
T4	1 (0.6)	0 (0)	
Unknown	13 (7.2)	3 (2.7)	
Grade			**0.026** ^2^
I	10 (5.6)	8 (7.1)	
II	91 (50.6)	40 (35.4)	
III	74 (41.1)	64 (56.6)	
Unknown	5 (2.8)	1 (0.9)	
Histological type			0.227^2^
Ductal	132 (73.3)	92 (81.4)	
Lobular	30 (16.7)	11 (9.7)	
Other	15 (8.3)	10 (8.8)	
Unknown	3 (1.7)	0 (0)	
ER expression			0.609^3^
Under 1%	24 (13.3)	18 (15.9)	
1% or more	154 (85.6)	95 (84.1)	
Unknown	2 (1.1)	0 (0)	
PR expression			0.1^3^
Under 1%	40 (22.2)	36 (31.9)	
1% or more	137 (76.1)	77 (68.1)	
Unknown	3 (1.7)	0 (0)	
HER2 amplification			**<0.001** ^3^
Positive	31 (17.2)	40 (35.4)	
Negative	146 (81.1)	73 (64.6)	
Unknown	3 (1.7)	0 (0)	
Ki-67 expression			0.068^3^
0–15%	49 (27.2)	22 (19.5)	
16–100%	116 (64.4)	91 (80.5)	
Unknown	15 (8.3)	0 (0)	
Molecular subtype			**0.013** ^2^
Luminal A	28 (15.6)	9 (8.0)	
Luminal B (HER2 negative)	98 (54.4)	55 (48.7)	
Luminal B (HER2 positive)	24 (13.3)	31 (27.4)	
Her2 positive	7 (3.9)	9 (8.0)	
TNBC	14 (7.8)	9 (8.0)	
Unknown	9 (5.0)	0 (0)	
Type of surgery			0.155^2^
Mastectomy	54 (30.0)	28 (24.8)	
Resection	122 (67.8)	85 (75.2)	
Unknown	4 (2.2)	0 (0)	
Adjuvant chemotherapy			**0.004** ^2^
Anthracycline and taxane-based	148 (82.2)	108 (95.6)	
Taxane-based	15 (8.3)	0 (0)	
Other	11 (6.1)	5 (4.4)	
Unknown	6 (3.3)	0 (0)	

ER: estrogen receptor; PR: progesterone receptor; TNBC: triple-negative breast cancer.

1 Mann–Whitney *U* test.

2 Chi-square test.

3 Fisher’s exact test.

Bolded values indicate statistically significant *p*-values.

### Comparison between the HRQoL of 0–3 months and 0–6 months in the pandemic era

**Figure 1:**
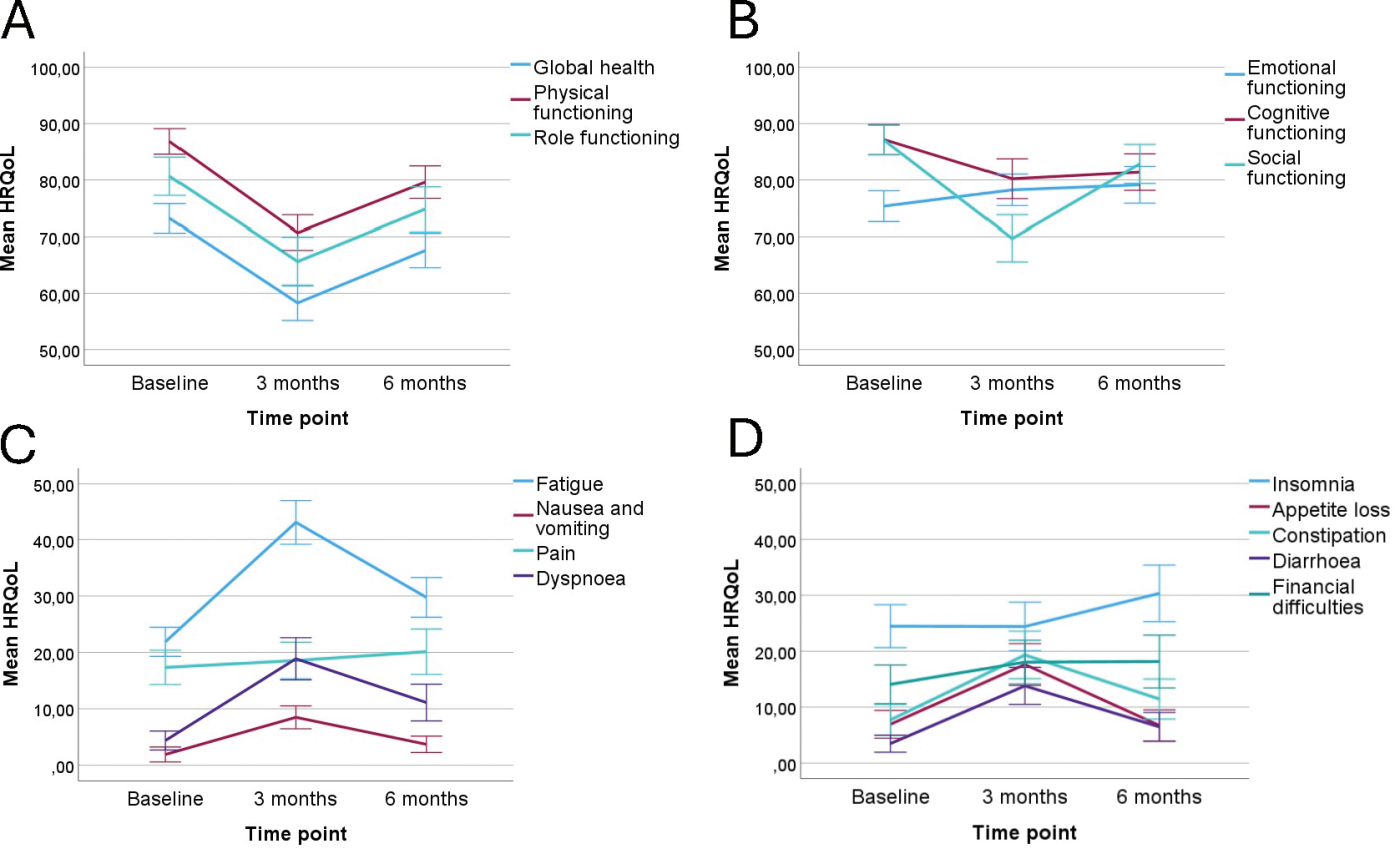
(A–D) The mean EORTC QLQ-C30 questionnaire scores for the functional and symptom scales at three time points for the patients treated in the pandemic era with 95% confidence intervals. The statistically significant results are presented.

The results of the HRQoL questionnaires and comparison between different time points in the pandemic era are shown in Table 2 and Figure 1.

**Table 2:** Comparison of the EORTC QLQ-C30 scores in the pandemic era between 0 and 3 months and 0 and 6 months.

EORTC QLQ-C30 subscale	Pandemic era, baseline 0 months	Pandemic era, 3 months	Pandemic era, 6 months	*p* (0–3 months)	*p* (0–6 months)
	Mean score (SD), *n*	Mean score (SD), *n*	Mean score (SD), *n*		
**Global health**					
Global health status	73.23 (17.32), *174*	58.28 (19.99), *160*	67.58 (18.04), *137*	**<0.001**	**<0.001**
**Functional scales**					
Physical functioning	86.91 (15.13), *175*	70.71 (19.94), *160*	79.61 (17.05), *137*	**<0.001**	**<0.001**
Role functioning	80.76 (22.56), *175*	65.63 (27.64), *160*	74.82 (23.49), *137*	**<0.001**	**0.007**
Emotional functioning	75.34 (18.26), *174*	78.23 (17.90), *160*	79.14 (19.26), *137*	0.142	**0.015**
Cognitive functioning	87.16 (17.87), *174*	80.21 (22.60), *160*	81.39 (19.18), *137*	**<0.001**	**<0.001**
Social functioning	87.07 (17.48), *174*	69.69 (26.56), *160*	82.85 (20.41), *137*	**<0.001**	**0.020**
**Symptom scales**					
Fatigue	21.88 (17.28), *175*	43.06 (24.77), *160*	29.72 (20.50), *137*	**<0.001**	**<0.001**
Nausea and vomiting	1.89 (8.88), *175*	8.44 (12.95), *160*	3.65 (8.51), *137*	**<0.001**	**0.001**
Pain	17.33 (20.27), *175*	18.75 (20.79), *160*	19.98 (23.64), *136*	0.233	**0.013**
Dyspnea	4.36 (11.27), *175*	18.87 (23.88), *159*	11.27 (19.13), *136*	**<0.001**	**<0.001**
Insomnia	24.24 (25.56), *175*	24.53 (27.42), *159*	30.17 (29.40), *137*	0.278	**0.003**
Appetite loss	6.82 (16.45), *175*	17.61 (23.66), *159*	6.57 (16.09), *137*	**<0.001**	0.856
Constipation	7.58 (18.33), *175*	19.17 (26.84), *160*	11.52 (20.84), *136*	**<0.001**	**0.013**
Diarrhea	3.43 (10.15), *174*	13.63 (20.95), *159*	6.37 (14.92), *136*	**<0.001**	**0.048**
Financial difficulties	13.98 (23.00), *173*	17.82 (24.81), *159*	18.02 (27.55), *135*	**0.003**	**0.027**

*P*-values from the Wilcoxon signed rank test.

SD: Standard deviation; *n*: number of respondents.

Bolded values indicate statistically significant *p*-values.

At 3 months, during the adjuvant chemotherapy, the patients reported a significant decrease in their EORTC QLQ-C30 functional subscales regarding global health status, physical, role, cognitive and social functioning. For example, the mean score of global health status at baseline was 73.23 and at 3 months 58.28, and the mean score of social functioning at baseline was 87.07 and 69.69 at 3 months. At 6 months, all the other functional scales still deteriorated, but emotional functioning increased compared to the baseline results.

From the individual symptoms, fatigue, nausea and vomiting, dyspnea, appetite loss, constipation, diarrhea and financial difficulties increased significantly from baseline to 3 months. As examples, fatigue increased from 21.88 at baseline to 43.06 at 3 months, and dyspnea from 4.36 to 18.87. Only appetite loss returned to the baseline level at 6 months, and other scales remained deteriorated even after the treatment at 6 months.

Insomnia and pain did not change during the chemotherapy treatment but declined at 6 months compared to the baseline results.

### Comparison of HRQoL between the COVID-19 pandemic and the pre-pandemic eras

**Figure 2:**
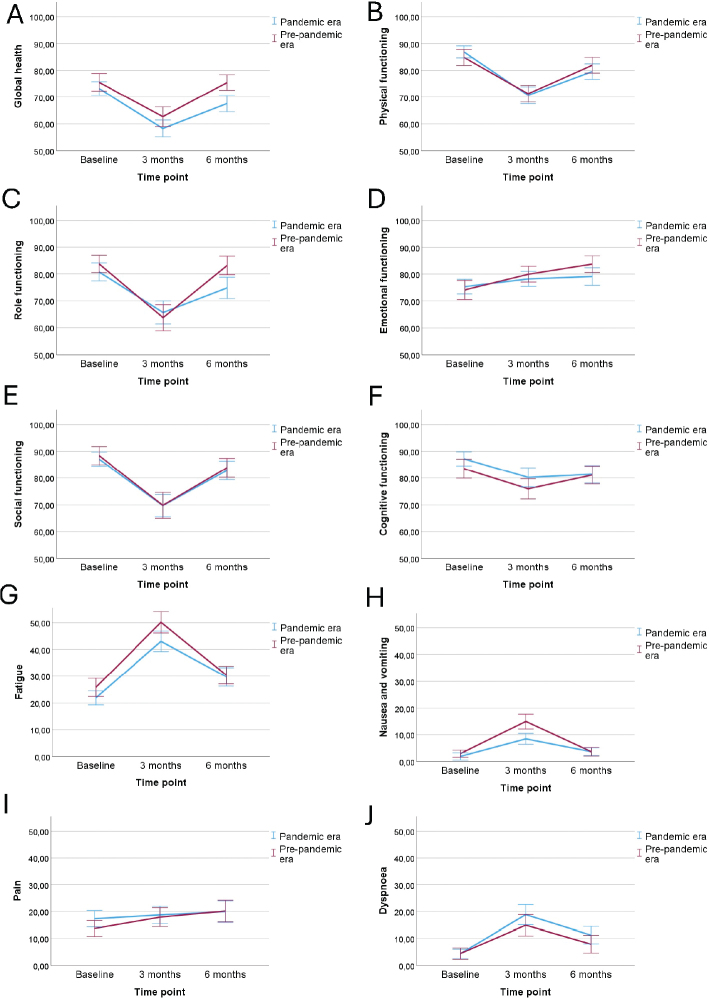
(A–J) The mean EORTC QLQ-C30 scores (with 95% confidence interval levels) between the patients in the pandemic and in pre-pandemic era for functional scales and for symptom scales fatigue, nausea and vomiting, pain and dyspnea.

**Figure 3:**
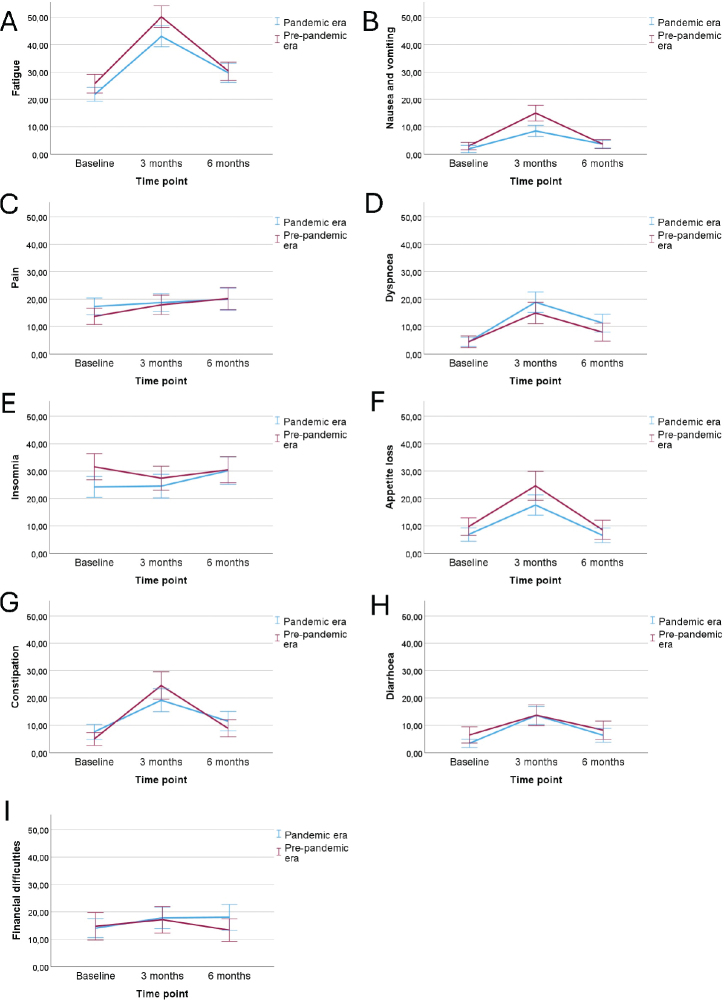
(A–I) The mean EORTC QLQ-C30 scores (with 95% confidence interval levels) between the patients in pandemic and in pre-pandemic era for the rest of the symptom scales.

The results from the EORTC QLQ-C30 questionnaires collected at different time points from the pandemic and pre-pandemic cohorts are found in Table 3 and in Figures 2–3.

**Table 3:** The mean EORTC QLQ-C30 scores in the pre- and pandemic era.

EORTC QLQ-C30 subscale	Pandemic era Mean (SD)	Pre-pandemic era Mean (SD), *n*	*p*
Baseline			
**Global health**			
Global health status	73.23 (17.32)	75.59 (17.56), *113*	0.261
**Functional scales**			
Physical functioning	86.91 (15.13)	84.88 (16.23), *112*	0.238
Role functioning	80.76 (22.56)	83.78 (17.32), *113*	0.614
Emotional functioning	75.34 (18.26)	74.12 (19.04), *113*	0.685
Social functioning	87.07 (17.48)	88.39 (18.15), *112*	0.303
Cognitive functioning	87.16 (17.87)	83.48 (19.22), *113*	0.068
**Symptom scales**			
Fatigue	21.88 (17.28)	25.79 (18.34), *112*	0.962
Nausea and vomiting	1.89 (8.88)	2.95 (7.12), *113*	**0.038**
Pain	17.33 (20.27)	13.72 (15.94), *113*	0.262
Dyspnea	4.36 (11.27)	4.42 (11.36), *113*	0.974
Insomnia	24.24 (25.56)	31.56 (25.53), *113*	**0.009**
Appetite loss	6.82 (16.45)	9.73 (17.07), *113*	0.065
Constipation	7.58 (18.33)	5.01 (12.77), *113*	0.375
Diarrhea	3.43 (10.15)	6.49 (15.97), *113*	0.101
Financial difficulties	13.98 (23.00)	14.75 (27.07), *113*	0.749
Three months			
**Global health**			
Global health status	58.28 (19.99)	62.69 (19.17), *107*	0.083
**Functional scales**			
Physical functioning	70.71 (19.94)	71.28 (16.37), *107*	0.835
Role functioning	65.63 (27.64)	63.71 (25.27), *107*	0.441
Emotional functioning	78.23 (17.90)	79.98 (15.22), *107*	0.617
Social functioning	69.69 (26.56)	69.78 (25.40), *107*	0.892
Cognitive functioning	80.21 (22.60)	75.94 (19.53), *106*	**0.021**
**Symptom scales**			
Fatigue	43.06 (24.77)	50.16 (20.61), *107*	**0.009**
Nausea and vomiting	8.44 (12.95)	14.95 (14.83), *107*	**<0.001**
Pain	18.75 (20.79)	17.91 (18.41), *107*	0.994
Dyspnea	18.87 (23.88)	14.95 (20.60), *107*	0.239
Insomnia	24.53 (27.42)	27.41 (22.81), *107*	0.144
Appetite loss	17.61 (23.66)	24.61 (27.60), *107*	**0.034**
Constipation	19.17 (26.84)	24.53 (26.15), *106*	**0.041**
Diarrhea	13.63 (20.95)	13.71 (19.93), *107*	0.844
Financial difficulties	17.82 (24.81)	17.13 (25.23), *107*	0.732
Six months			
**Global health**			
Global health status	67.58 (18.04)	75.48 (15.06), *105*	**<0.001**
**Functional scales**			
Physical functioning	79.61 (17.05)	81.97 (15.27), *105*	0.335
Role functioning	74.82 (23.49)	83.17 (17.83), *105*	**0.007**
Emotional functioning	79.14 (19.26)	83.73 (15.85), *104*	0.088
Social functioning	82.85 (20.41)	83.81 (18.06), *104*	0.938
Cognitive functioning	81.39 (19.18)	81.11 (16.99), *105*	0.626
**Symptom scales**			
Fatigue	29.72 (20.50)	30.37 (16.97), *105*	0.574
Nausea and vomiting	3.65 (8.51)	3.65 (8.33), *105*	0.998
Pain	19.98 (23.64)	20.19 (20.86), *104*	0.566
Dyspnea	11.27 (19.13)	7.94 (17.00), *105*	0.112
Insomnia	30.17 (29.40)	30.48 (24.51), *105*	0.672
Appetite loss	6.57 (16.09)	8.57 (17.92), *105*	0.275
Constipation	11.52 (20.84)	8.89 (16.19), *105*	0.534
Diarrhea	6.37 (14.92)	8.25 (17.16), *105*	0.407
Financial difficulties	18.02 (27.55)	13.33 (20.98), *105*	0.372

*P*-values from the Mann–Whitney U test.

SD: Standard deviation; *n*: number of respondents (for pre-pandemic patients in Table 2).

Bolded values indicate statistically significant *p*-values.

Before the start of the chemotherapy, patients in the pandemic era reported less nausea and vomiting (mean scores 1.89 and 2.95; *p* = 0.038) and insomnia (mean scores 24.24 and 31.56; *p* = 0.009) than the patients in the pre-pandemic period, but no differences were observed in the other subscales at baseline.

At 3 months, the patients treated in the pandemic era reported better cognitive functioning (mean scores 80.21 and 75.94; *p* = 0.021) and less fatigue (mean scores 43.06 and 50.16; *p* = 0.009), nausea and vomiting (mean scores 8.44 and 14.95; *p* < 0.001), appetite loss (mean scores 17.61 and 24.61; *p* = 0.034) and constipation (mean scores 17.61 and 24.61; *p* = 0.041) than the patients in the pre-pandemic era.

At 6 months, the patients in the pandemic cohort reported lower global health status (*p* < 0.001) and role functioning (*p* = 0.007) than the patients in the pre-pandemic cohort. The other subscales were similarly distributed between the groups.

## Discussion

This observational, multicenter study investigated the HRQoL of adjuvant chemotherapy receiving early-stage breast cancer patients during the COVID-19 pandemic. The study compared the results with the patients who participated in the prospective trial just before the pandemic in a similar setting. Most EORTC QLQ-C30 subscales in the patients treated in the pandemic era deteriorated statistically significantly from baseline to 3 months and remained declined after the adjuvant chemotherapy at 6 months. Surprisingly, the patients were performing better according to several subscales during the pandemic than in the pre-pandemic era.

In our study, the functional scales (the global health status, cognitive, physical, social and role functioning) deteriorated during the pandemic era and did not return to baseline levels at the 6-month follow-up. Cognitive functioning at 6 months remained worse than before the chemotherapy. Prior studies have shown that cognitive impairment is a common consequence of chemotherapy in breast cancer patients [24], and it can last even 6 months after completing taxane-based chemotherapy in these patients [15]. Consistent with our findings, experiences of breast cancer patients during the COVID-19 pandemic led to worsened cognitive and emotional health outcomes [5]. Our study on the pandemic cohort revealed a decline in HRQoL both during and after chemotherapy, compared to patients treated before the pandemic. In a Norwegian study, the QoL of the patients did not significantly decline during the pandemic as it remained consistently lower than that of healthy controls, but stable across different pandemic phases [20]. In that study, specific groups such as younger women with children were particularly affected. However, the above-mentioned studies involved a heterogeneous group of breast cancer patients and survivors with varying treatment modalities and disease phases, whereas our study concentrated on early breast cancer patients receiving adjuvant chemotherapy during the COVID-19 pandemic.

Our patients may have ignored or underestimated adjuvant chemotherapy-related symptoms when the focus was on the COVID-19 restrictions. This could account for a relatively good HRQoL during the pandemic in our study. Typically, by the time of the 6-months’ HRQoL survey, after completing the radiotherapy, the employed patients return to work. The daily routine of work after the chemotherapy period might explain the still lower levels of scores in subscales such as physical functioning compared to baseline.

Of the patients in the pandemic cohort, 82% received anthracycline and taxane-based treatment, compared with 96% of the patients in the pre-pandemic era. Anthracycline-based treatment is highly emetogenic; it can cause loss of appetite and the need for antiemetic therapy, which can cause obstipation. This may at least partly explain the unexpected differences at 3 months in the side-effect scales between the pre-pandemic and pandemic cohorts. Other clinical features of the pandemic cohort patients compared to the pre-pandemic patients were not significantly different, except for lower number of patients with HER2 amplification and higher BMI, which are not likely to lead to different experience of the HRQoL during the adjuvant chemotherapy.

At 6 months, the global health status and the role functioning were still worse in the pandemic cohort than in the pre-pandemic cohort, while the other subscales were equally contributed. As these EORTC QLQ-C30 items specifically describe the limitations in work and other activities and in the experienced overall HRQoL and health, these results are likely not related to cancer survivorship, but rather to the COVID-19 measures and the pandemic and its restrictions.

In other studies, during the COVID-19 pandemic, patients with breast cancer and gynecological malignancies reported significant increases in anxiety, depression and mood swings [25] and patients with various cancer types reported increased anxiety and depression [26]. Interestingly, in our cohort of pandemic patients, the emotional functioning at 6 months was significantly improved compared to the baseline, and no differences between the pandemic and pre-pandemic patient cohorts were observed regarding emotional functioning (tension, worries, depression, irritation) at any of the studied time points. This may be attributed to the accomplished adjuvant chemotherapy with no pandemic-related delays, causing relief and no extra worries for the patients.

This is the first study to focus on the HRQoL of breast cancer patients receiving adjuvant chemotherapy during the COVID-19 pandemic. In a previous study consisting of breast cancer and ductal carcinoma in situ patients, several EORTC QLQ-C30 parameters were improved during the pandemic compared to the pre-pandemic era from same breast cancer patients and survivors, including quality of life, physical, role, cognitive and social functioning [27]. In that study, with only 5% of patients under active treatment, only social functioning deteriorated in actively (with chemo- or radiotherapy) treated patients during the pandemic [27]. Similar to our findings, the global health status of early and advanced breast cancer patients deteriorated during the COVID-19 pandemic, when compared to pre-pandemic non-cancerous general population reference data [28]. As limitations, in these studies, the pandemic patient material consisted of a heterogeneous breast cancer population with no specific classification of cancer type, clinical parameters or treatment.

During the COVID-19 pandemic worldwide, more de novo stage IV breast cancer cases were found [29], lymph node metastases were more frequent [30, 31] and greater tumor sizes were observed [31]. In Finland, the number of new malignancies declined in 2020 compared to the year before but rose during the second half of 2020 almost to the same level as in 2019 [32]. Nevertheless, in our study, the nodal status and the tumor size did not differ between the pre-pandemic and the pandemic cohorts. Also, the HRQoL results differed only moderately, and in many subscales in favor of pandemic time. One could discuss the possibility that the COVID-19 pandemic did not heavily affect the patient experience of cancer care in Finland. Longer follow-up from our cohort and other sources will show possible differences in the survival rates.

The pre-pandemic comparison population from the same country, with the same EORTC QLQ-C30 questionnaire time points in relation to adjuvant chemotherapy, and the collection from the same country just before the pandemic can be considered as the strengths of the current study. The pre-pandemic cohort consisted of patients in the prospective BOUNCE study [21], which was designed to predict resilience after breast cancer treatments. Importantly, the participants did not receive any specific intervention to support their well-being outside the usual clinical practice neither in our or in the BOUNCE study. Both prospective cohorts had rather similar baseline characteristics, with the main exception being the more HER2-positive tumors in the pre-pandemic cohort. The response rates for the questionnaires were of good value, for the pandemic patients 97% at the baseline, 88% at 3 months and 75% in 6 months, whereas for pre-pandemic patients they were 99% at the baseline, 94% at 3 months and 92% at 6 months. These response rates should not be considered as comparable due to different study designs.

As the drawbacks in the current study, a rather small number of all the newly diagnosed breast cancer patients participated in this study, and due to this, we were not able to formally evaluate the minimal important difference (MID) of HRQoL in a reliable way. Again, recent evidence suggests that MID values vary across cancer types, cohorts and QoL domains, typically ranging from 4 to 10 points in breast cancer populations [33]. In our study, the differences observed between groups were generally small – statistically significant contrasts ranged from 5 to 10 points, while non-significant differences were usually below 2 points, indicating that the clinical relevance of these findings is probably limited. The patients treated in five different hospitals in Finland may lead to more heterogeneity of the results, although the early-stage breast cancer guidelines in Finland are uniform, all the hospitals were University Hospitals and Finnish cancer care is almost solely based on the public sector. As it was not recorded how many patients were offered to participate in the study, we acknowledge the fact that selection bias is possible in this study, as it is in all HRQoL studies. Other limitations of our study design are the use of only the EORTC QLQ-C30 questionnaire and a follow-up of only 6 months, but again, the used questionnaire is validated and widely used in the breast cancer literature. As an inherent bias in all prospective HRQoL studies, patients with more stressful baseline life situations may have dropped out of the study more often.

## Conclusion

The main novelty of this study is evaluating the HRQoL of breast cancer patients receiving adjuvant chemotherapy during the COVID-19 pandemic and comparing the results to a very similar cohort just before the pandemic. Many HRQoL subscales for breast cancer patients treated during the COVID-19 pandemic deteriorated at 3 months and did not reach the baseline level at 6 months. The self-reported well-being of breast cancer patients in the pandemic era during the adjuvant chemotherapy was moderately better in comparison to the pre-pandemic era, although changes in the treatment might partly explain this. After the chemotherapy, the patients treated in the pandemic era, showed a decline in HRQoL compared to the pre-pandemic era. Although the COVID-19 pandemic caused distress for cancer patients, they might have reported fewer symptoms due to better resilience to their cancer treatment during the pandemic. Decreased global health and role functioning after the treatment may be a cause of the pandemic restrictions and not related to the chemotherapy. Consequently, COVID-19 did not heavily affect the patient experience of cancer care in Finland. These findings underscore the importance of including mental health services in future public health crises.

## Data Availability

All data generated or analyzed during this study are included in this published article. The raw data used in the analyses of the current study are not publicly available due to legislative issues concerning the privacy of the patients.
